# Dim-light colour vision in the facultatively nocturnal Asian giant honeybee, *Apis dorsata*

**DOI:** 10.1098/rspb.2023.1267

**Published:** 2023-08-09

**Authors:** Sajesh Vijayan, G. S. Balamurali, Jewel Johnson, Almut Kelber, Eric J. Warrant, Hema Somanathan

**Affiliations:** ^1^ School of Biology, IISER-TVM Centre for Research and Education in Ecology and Evolution (ICREEE), Indian Institute of Science Education and Research Thiruvananthapuram, Thiruvananthapuram, Kerala, India; ^2^ Lund Vision Group, Department of Biology, University of Lund, Sölvegatan 35, Lund 22362, Sweden

**Keywords:** honeybee, apposition compound eye, nocturnal colour vision, pollination

## Abstract

We discovered nocturnal colour vision in the Asian giant honeybee *Apis dorsata—*a facultatively nocturnal species*—*at mesopic light intensities, down to half-moon light levels (approx. 10^−2^ cd m^−2^). The visual threshold of nocturnality aligns with their reported nocturnal activity down to the same light levels. Nocturnal colour vision in *A. dorsata* is interesting because, despite being primarily diurnal, its colour vision capabilities extend into dim light, while the ‘model’ European honeybee *Apis mellifera* is reported to be colour-blind at twilight. By employing behavioural experiments with naturally nesting *A. dorsata* colonies, we show discrimination of the trained colour from other stimuli during the day, and significantly, even at night. Nocturnal colour vision in bees has so far only been reported in the obligately nocturnal carpenter bee *Xylocopa tranquebarica.* The discovery of colour vision in these two bee species, despite differences in the extent of their nocturnality and the limitations of their apposition compound eye optics, opens avenues for future studies on visual adaptations for dim-light colour vision, their role in pollination of flowers at night, and the effect of light pollution on nocturnal activity in *A. dorsata,* a ubiquitous pollinator in natural, agricultural and urban habitats in the Asian tropics and sub-tropics.

## 1. Introduction

Nocturnal and crepuscular animals experience rapidly fluctuating ambient light environments. Under these conditions, objects are more reliably perceived by visual systems that possess the ability for colour vision than by those that do not [1,2]. That said, humans are incapable of using colour as a reliable visual signal at night, and switch from cone-based photopic vision that can detect colour to mesopic vision that depends on both rods and cones until half-moon light levels, below which humans depend on rod-based scotopic vision that can only distinguish intensity gradients [3,4]. Therefore, unsurprisingly, nocturnal vision was thought to be mainly achromatic and nocturnal pollination by flower visitors was believed to be mediated mainly by olfaction, since most night-blooming flowers are highly fragrant [5,6]. That view changed with the discovery of colour vision in the nocturnal elephant hawkmoth *Deilephila elpenor* [7]. Subsequent confirmation from two other hawkmoths and a carpenter bee suggests that the ability to detect objects by the spectral component of light is an important aspect of dim-light vision in nocturnal flower-visiting insects as well as for nocturnal pollination [1,7–9].

Seeing colour in the dark requires special visual adaptations*—*morphological, physiological and possibly neuronal [1,2]. In hawkmoths, this includes having superposition compound eyes whose low F-number optics produce a significantly brighter image [1], and neural summation strategies that increase the signal-to-noise ratio of vision [7,10–13]. Interestingly, apart from hawkmoths, dim-light colour vision is also found in the nocturnal carpenter bee *Xylocopa tranquebarica* despite this bee—like all other bees—possessing apposition compound eyes better suited for vision in bright light [5,9,14]. Although the apposition design severely reduces the number of photons absorbed by each photoreceptor, the large eyes of this carpenter bee, together with large facet lenses and wide rhabdoms, all increase visual sensitivity. Neuronal adaptations might increase sensitivity further [9], as found in another nocturnal bee, *Megalopta genalis* [15]. In addition to insects, nocturnal colour vision has been shown in three vertebrate species—the nocturnal helmet gecko (*Tarentola chazaliae* [16]) and two amphibians (*Bufo bufo* and *Rana temporaria* [17], which possess a dual-rod visual system).

Honeybees are among the most identifiable pollinator groups [18] and their role as pollinators and their importance in maintaining biodiversity has been extensively studied over several decades [19–21]. Numerous studies, spurred by the discovery of colour vision about a century ago in the European subspecies of the western honeybee *Apis mellifera* (hereon referred to as the European honeybee) [22], have established this species as an important model for understanding invertebrate colour vision and visual ecology in general [23–25]. Colour vision in honeybees is realized through three spectral classes of photoreceptors which are maximally sensitive to the ultraviolet (344 nm), blue (436 nm) and green (556 nm) regions of the spectrum [26]. The number and sensitivities of photoreceptors are highly conserved across hymenopterans, with floral colour detection being one of several evolutionary drivers [27–29]. While floral colour and insect colour vision exhibits ‘matching’ [30], the precise evolutionary relationship between the two is still debated [29]. The apposition optics of their visual systems have evolved to provide high spatial acuity and colour perception under brightly lit diurnal conditions [24,31]. Not surprisingly, colour perception in *A. mellifera* reduces drastically at light levels corresponding to dim twilight and moonlit nights (10^−1^–10^−2^ cd m^−2^), below which they switch to using achromatic cues [32]. This inability to detect colour in dim-light could be a major reason why most honeybees limit their activity to diurnal periods, with the only exceptions being facultative nocturnality in the African sub-species of *A. mellifera* (*A. m. adansonii*) and the Asian giant honeybee (*Apis dorsata*) [33–35].

**Figure 1.**
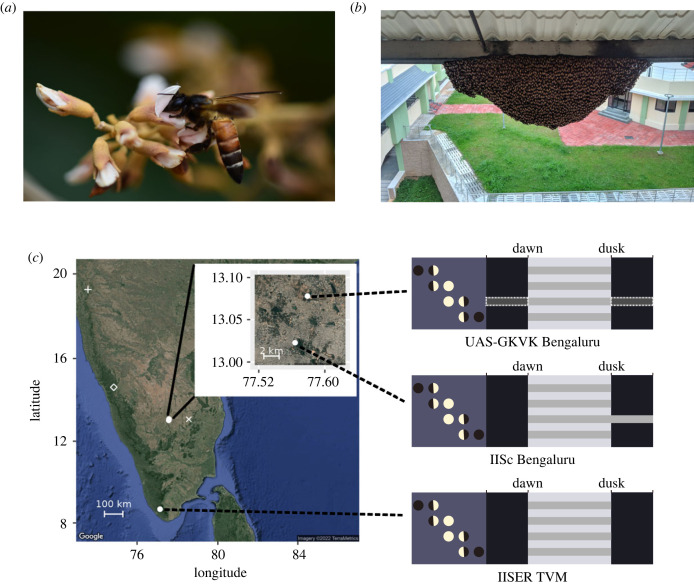
(*a,b*) An *Apis dorsata* forager and the experimental colony at IISER-TVM. *Apis dorsata* nests consist of a single comb built in the open. (*c*) Map of southern India showing locations where the experiments were carried out: IISER-TVM, IISc Bengaluru and UAS-GKVK Bengaluru (denoted by white circular spots). The foraging activity of *A. dorsata* workers across the four lunar quarters during our experiments are denoted by the light grey horizontal bars and the nocturnal period is denoted by the black areas. Nocturnal activity was observed only at IISc Bengaluru, although earlier studies [38,39] have reported nocturnal activity at UAS-GKVK Bengaluru (dark grey bars with dotted-white outline). Night activity in *A. dorsata* has also been observed at Kaigal (by Kavya Mohan; cross), at Sirsi (diamond; [34]) and at Bhimashankar (plus symbol; [35]).

*Apis dorsata* Fabricius 1793 is an open-nesting honeybee with bees forming broad ‘curtains’ covering the comb [36,37] (figure 1*a,b*). They are known to be active under bright moonlight conditions (half-moon and brighter [34,38]) and when specific floral resources are available [35]. Despite being one of the largest honeybees, they are much smaller when compared with the nocturnal carpenter bee, and hence likely to be severely limited in terms of the visual adaptations required for nocturnal colour vision [35]. Besides their facultative nocturnal behaviour, *A. dorsata* are open-nesting, building hives on tall cliffs, trees and on high-rise buildings in urban areas [40–42], which makes this species an ideal system to study the role of colour vision*,* especially the effect of increasing artificial light at night on colour perception and visual behaviour [43,44].

The objective of this study was to test for diurnal and nocturnal colour vision in the facultatively nocturnal *A. dorsata* using behavioural experiments with wild, naturally occurring colonies. We first tested whether these bees possess colour vision in bright daylight by training them to associate food with a coloured target and then tested their ability to detect this coloured target amidst grey targets of varying intensities, as well as to discriminate the learned target from targets of other colours. Next, in a similar manner, we tested their ability to detect coloured targets at night and determined the potential threshold of colour vision in this bee.

## 2. Methods

### (a) Apis dorsata *colonies*

The diurnal colour vision experiments were carried out from August 2019 to December 2021 on the roof of the Biological Sciences building at the IISER Thiruvananthapuram campus, India (8.68°N, 77.14°E) (figure 1*c*). The nocturnal colour vision experiments were carried out from March to May 2022 on the rooves of the Centre for Ecological Sciences building at the Indian Institute of Science, India (13.02°N, 77.56°E) and the University of Agricultural Sciences, GKVK—Bengaluru, India (13.08°N, 77.58°E) (figure 1*c*). All experiments were carried out using workers from naturally nesting colonies of *A. dorsata*.

We recruited bees to a black feeder which contained sucrose solution (30% w/v, henceforth referred to as the recruitment feeder) that was placed approximately 5 m away from the colonies. After a steady number of foragers were observed to visit the feeder, the sucrose concentration was reduced to 5–10% (w/v) to avoid overcrowding. The feeder was gradually moved towards the experimental arena which was located 10–20 m away from the colonies.

### (b) Experimental stimuli and colour modelling

**Figure 2.**
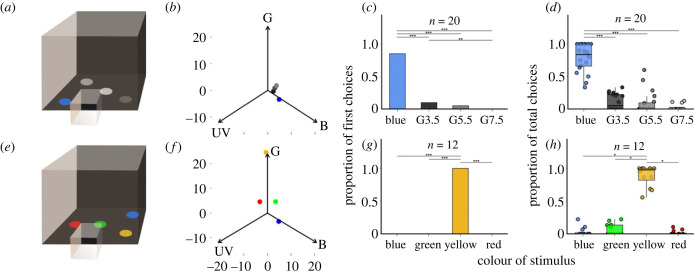
Diurnal colour vision in *Apis dorsata*. (*a*) The set-up used for the colour discrimination assays using blue and three grey stimuli (details in the main text). (*b*) Stimuli in bee colour space as visualized using the RNL model [43]. (*c,d*) After training, bees were tested for their ability to discriminate the training colour within an array comprising four stimuli: the trained blue and three grey stimuli (G3.5, G5.5 and G7.5). The first and total choices of 20 bees were recorded (*c,d,* respectively). (*e*) Colour discrimination assays were also carried out in the same arena using another set of 12 bees trained to a yellow colour and tested on an array consisting of yellow, blue, green and red target stimuli. (*f*) The distribution of the four coloured stimuli in bee colour space using the RNL model. With both sets of bees that were trained to either blue or yellow, the first landings of trained bees were predominantly to the trained colour (*c,g*). The upper horizontal bound of the box corresponds to the proportion of bees that visited each stimulus. The horizontal lines and the asterisk above the boxes correspond to significant pair-wise differences in the visits between stimuli as estimated from a post-hoc *χ*^2^ comparison with Bonferroni correction; **p < 0.01, ***p < 0.001. Bees also made more total visits to the trained stimuli (*d,h*). The hinges (horizontal bounds of the box) correspond to the interquartile range (IQR), the bold horizontal lines correspond to the medians, the whiskers denote the upper/lower hinge ± 1.5xIQR, the filled circles correspond to individual data points and those outside the whiskers represent outliers. The horizontal lines and the asterisk above the boxes correspond to significant differences in the visits between stimuli as estimated from a post-hoc Wilcoxon signed rank test on paired samples; *p < 0.05, ***p < 0.001.

All diurnal experimental targets were 8 cm diameter laminated discs made from papers manufactured by Color-aid Corporation USA, and presented on a horizontal black background. We measured the reflectance spectra of the stimuli and background using an Ocean Insight Ocean-HDX-UV-VIS spectrophotometer connected to an Ocean Optics PX-2 pulsed Xenon light-source (electronic supplementary material, figure S1). The bees were trained to a blue target (B-Hue) and tested in an array of blue and three grey targets of varying intensities (Gray3.5, Gray5.5 and Gray7.5, referred to as G3.5, G5.5 and G7.5, respectively) (figure 2*a*). We also replicated this after training worker bees on a horizontal array to a yellow target (Yw-Hue) and testing them against yellow and three grey targets (Gray5, Gray7 and Gray9 (electronic supplementary material, figure S2*a*)). In the colour discrimination assay, bees were trained to blue (B-Hue) and tested in a four-colour vertical array consisting of the trained blue, green (G-Hue), yellow (Yw-Hue) and red (Rw-Hue) targets, respectively (figure 2*e*).

At night, the bees became demotivated when we switched from the black training feeder to flat circular blue targets in darker conditions. We therefore replaced the black feeder with a blue training feeder (11 cm diameter, 6 cm height) containing 30% sucrose (w/v), approximately 15 min after sunset and allowed the bees to continuously visit the feeder. We tested nocturnal colour vision on an array of unrewarded feeders covered with the blue and the grey stimuli (B-Hue, G3.5, G5.5 and G7.5). The stimuli were presented against a black background in all experiments. We modelled the stimuli in the honey bee visual space using the receptor noise limited (RNL) model [45] in the *colourvision* and *pavo* packages in R [46,47] and the colour hexagon model [48] in the *colourvision* package. Along with the behavioural assays, we sought to establish that *A. dorsata* uses colour information at night by showing that the trained colour was chromatically different from the background at all light levels, and that the relative brightness, given by the green receptor contrast [14], was an unreliable cue [49]. We modelled the stimuli under three light levels (electronic supplementary material, figure S1*b*): bright daylight (D65 spectrum from [46]), moonlight (spectrum from [50]) and twilight conditions (sun 6.5° below horizon; spectrum from [51]), using the photoreceptor sensitivity curves and photoreceptor noise for *A. mellifera*.

### (c) Behavioural assays

#### (i) Diurnal assays

The arena for the colour vision experiments in bright daylight consisted of a flight cage of dimensions 60 cm × 60 cm × 60 cm, made from 2 mm thick UV-transparent acrylic sheets (figure 2*a,e,* electronic supplementary material, figure S2*a*). Bees entered the arena through an entrance corridor made of the same material (20 cm × 20 cm × 20 cm). The traffic of bees was controlled using two gates on either end of the corridor that enabled bees to individually enter the arena during training and testing.

We tested whether *A. dorsata* has colour vision by using two slightly modified versions of the experimental paradigm established by Karl von Frisch [22]. In the *colour detection assay*, we tested if the bees could detect the trained colour amidst grey stimuli of varying intensities. In the *colour discrimination assay*, we estimated the ability of bees to detect the trained stimulus from other chromatic stimuli. In both assays, worker bees were trained to fly into the arena through the corridor by moving the black feeder gradually inside the arena, and they were allowed to leave from the top face of the arena after feeding (figure 2*a,e*, electronic supplementary material, figure S2*a*). We began the training once 5–10 bees returned to the black feeder inside the arena. Individual bees were identified by marking them with acrylic paints (Camlin Fabrica, India) on the thorax and/or abdomen while they fed from the coloured stimulus. This enabled us to distinguish individual foragers and track the number of training trials each bee received.

Marked bees fed *ad libitum* from the stimulus associated with a sucrose reward (20% w/v) before leaving the arena. This comprised one bout. The training comprised five bouts, between which the stimulus and black background were wiped with 70% ethanol and dried or replaced with a clean stimulus/background. The position of the stimulus was randomized between bouts. The bees were tested on a random array comprising the training stimulus and three other stimuli. For the colour detection tests these were grey targets. In the colour discrimination tests, these other targets were a red, a green and a blue stimulus. During training the sucrose reward was presented on a small petri dish placed on top of each stimulus target (and in a 200 µl micropipette tip at the centre of each target in the yellow-trained assays). All tests were unrewarded. A bee was considered to have made a choice if it landed on a stimulus. The first choice, and the total number of choices made across all stimuli, by each bee in 1 min were recorded by the observer. The tested bees were captured and sacrificed to avoid pseudoreplication.

#### (ii) Dim-light assays

**Figure 3.**
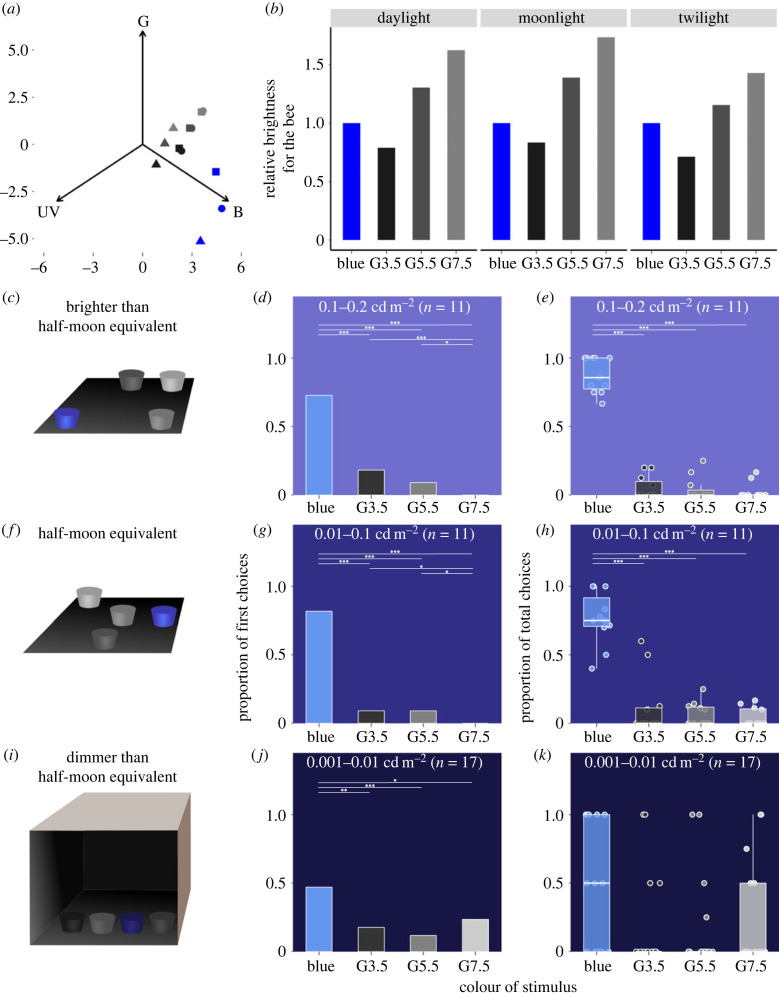
Nocturnal colour vision in *Apis dorsata*. (*a*) Stimuli in bee colour space estimated using the RNL model [45]. The points correspond to stimulus perception under daylight (filled triangles), moonlight (filled circles) and twilight conditions (filled squares). (*b*) The brightness of the stimuli in the test array relative to the brightness of the training stimulus (blue stimulus). The brightness is estimated as the output of the green receptor of honeybees. (*c,f,i*) The set-up used for each experiment (details in the main text). Bees (*n* = 39) were trained to a blue stimulus and their ability to detect the trained colour was tested within a target array consisting of four target stimuli: blue, G3.5, G5.5 and G7.5 under brighter and darker conditions. (*d,g,j*) *A. dorsata* showed a strong tendency to first land on the blue stimulus across all light levels. The upper horizontal bound of the box corresponds to the proportion of bees that visited each stimulus. (*e,h,k*) Proportion of total visits differed significantly between stimuli at all light levels (0.1–0.2 cd m^−2^: $\chi_{3}^{2}=128.1$, *p* < 0.001; 0.01–0.1 cd m^−2^: $\chi_{3}^{2}=174.3$, *p* < 0.001; 0.001–0.01 cd m^−2^: $\chi_{3}^{2}=28.7$, *p* < 0.001). The horizontal lines and the asterisk above the boxes correspond to significant pair-wise differences in the visits between stimuli as estimated from a post-hoc *χ*^2^ comparison with Bonferroni correction; *p < 0.05, **p < 0.01, ***p < 0.001. Bees made significantly more visits to the trained stimulus under brighter conditions (*e*) and (*h*), but this preference was not found under the dimmest condition (*k*). The hinges (horizontal bounds of the box) correspond to the IQR, the bold horizontal lines correspond to the medians, the whiskers denote the upper/lower hinge ± 1.5xIQR, the filled circles correspond to individual data points and those outside the whiskers represent outliers. The horizontal lines and the asterisk above the boxes correspond to significant differences in the visits between stimuli as estimated from a post-hoc Tukey HSD; ***p < 0.001.

Experiments at night were carried out in ambient light intensities corresponding to half-moon-equivalent (HME; 0.01–0.1 cd m^−2^), dimmer than HME (0.001–0.01 cd m^−2^), and brighter than HME (0.1–0.2 cd m^−2^). Training and testing at HME and brighter conditions were carried out on an array of targets arranged on a square base which the bees could freely access (figure 3*c,f*). The base was made of a cardboard sheet (60 cm × 60 cm) covered with a matte black vinyl sticker (LG hausys, LG) on which stimuli were presented in a randomized manner. Since natural luminance at the study site did not drop below HME, to test colour vision at dimmer light levels between 0.001 and 0.01 cd m^−2^, we covered the base with a cardboard box (60 cm × 60 cm × 60 cm; figure 3*i*). The inner faces of the arena were covered with black chart paper and the front was kept open to provide uninterrupted access to the bees during training and testing.

During overcast conditions, when urban lights lit the underside of clouds, the night-time luminance at the location where the dim-light colour vision experiments were being carried out was frequently greater than HME (0.01–0.1 cd m^−2^). We hence carried out these experiments at light levels corresponding to HME, and under brighter sky-glow conditions (0.1–0.2 cd m^−2^) in a single lunar cycle (9–22 April 2022). We used an open (uncovered) array for the experiments since most bees did not enter the set-up from the daylight experiments at lower light levels. Moreover, the ones that entered made no choice, became demotivated and failed to return for subsequent training. Hence bees could not be trained and tested individually. At onset of night (i.e. at the end of astronomical twilight), we marked the bees that were on the feeder with acrylic paint. This was considered as the first training, and the bees were allowed to train four more times before we replaced the blue feeder with the test array which comprised four empty (unrewarded) feeders covered in the blue or grey stimuli which were used for the colour detection assay in bright daylight. Coloured feeders similar to the blue training feeder were used instead of the flat circular targets used in the diurnal experiments since the bees were highly sensitive to the change in the experimental conditions and did not make any choice when the blue training feeder was switched with circular targets. The stimuli were presented against a black background in all experiments. The first choice by a bee and all choices made over a 1-min duration after the first choice were recorded by the observer, after which the bees were captured and sacrificed to avoid pseudoreplication.

Experiments at light levels below HME (0.001–0.01 cd m^−2^) were carried out by placing the array inside a box during twilight since the natural ambient light intensity at our study site was never lower than HME, and the bees were not motivated to enter the darkened set-up at night. The blue feeder was placed in the box approximately 15 min prior to sunset and the bees were marked once the activity dropped to approximately 30 bees at the feeder. The test array was placed in the set-up once the luminance inside the box dropped below 0.01 cd m^−2^. The first choice and all choices made by a bee over a 1-min duration after the first choice were noted using a pair of infra-red sensitive night-vision goggles (ATN NVG7). The bees were sacrificed after testing. The black background and stimuli were wiped clean with alcohol and allowed to dry after each training and test. The luminance for nocturnal experiments was measured using a Hagner ERP-105 Digital Photometer by holding the probe at a 45° angle against a horizontally placed white surface of known reflectivity (93.1%).

### (d) Statistical analyses

For the diurnal colour vision experiments, the effect of stimulus colour on the first choice made by bees (a landing was considered as a choice) was examined using a χ^2^-test, and pairwise differences between stimuli were estimated using χ^2^-tests with Bonferroni correction. The effect of stimulus colour on all choices made by a bee before flying out of the arena was quantified using the Friedman test, and pairwise comparisons between stimuli were carried out post-hoc using the Wilcoxon signed rank test on paired samples with Bonferroni correction. The effect of stimulus colour on the first choices made during dim-light tests was also estimated using a χ^2^-test and pairwise post-hoc comparisons were done using χ^2^-tests with Bonferroni correction.

Dim-light colour vision performance in *A. dorsata* at different ambient light intensities was compared using ANOVA. We first carried out a two-way ANOVA with stimulus colour, light levels and their interaction as fixed effects (model 1) and then we added bee identity as a random effect and carried out a repeated measures two-way mixed-effects ANOVA with the fixed-effects unchanged from model 1 (model 2) using the lme function from the *nlme* package [52]. The best model was selected on the basis of lowest AIC and pairwise differences were estimated using Tukey HSD.

All analyses were carried out in the R statistical programming environment (v. 4.1.3) [53] using the RStudio IDE (2022.02.3 Build 492) [54].

## 3. Results

### (a) Brightness of the stimulus is an unreliable cue

The perceptual distance needed to discriminate between stimuli in honey bee visual space as estimated from *Apis mellifera* under bright-light conditions is 2.3 RNL units [55,56], although finer discrimination of around 0.3 RNL units is achievable through differential training [57,58]. Similarly, the perceptual distance in colour hexagon space is between 0.01 and 0.02 hex units [57,58]. In our experiment, the training stimuli were discriminable from the background in all experiments and light conditions (RNL models for diurnal condition: figure 2*b,f*; electronic supplementary material, figure S2*b* and table S3; RNL models for dim-light conditions: figure 3*a*; electronic supplementary material, table S3; colour hexagon models: electronic supplementary material, figure S3*a* and table S3). At lower light intensities, additional noise in the form of photon shot noise can influence colour perception [2]. We used the *pavo* package to model the distance between stimuli in the RNL colour space under the three light conditions (electronic supplementary material, table S4). At lower light levels, the detection threshold is influenced by photon shot noise, which in turn translates to smaller distances between stimuli in the colour space. The model outputs using moonlight and late twilight spectra from the literature, and data valid for the *A. mellifera* visual system, returned very low values (less than 1 RNL unit) for all pairwise comparisons between the stimuli, suggesting poor colour detection ability under dim light (electronic supplementary material, table S4). Yet, in our experiment, *A. dorsata* workers were able to detect the blue stimulus at night (see below). This disparity between the model output and the bee's behaviour is explored in greater detail below (see Discussion). Importantly, we found that the relative brightness of the grey stimuli with respect to the blue stimulus fluctuated depending on the ambient light spectra (figure 3*b*; electronic supplementary material, figure S3*b* and table S3). Under daylight and moonlight conditions, the brightness of the blue stimulus was between the brightness values of the G3.5 and G5.5 stimuli. Under blue-shifted twilight, the blue stimulus is brighter and closer to G5.5 in brightness. When compared with the perceptual distances between stimuli, brightness is thus an unreliable cue for the detection of our stimuli, especially under the rapidly changing dim-light conditions in this study.

### (b) Diurnal colour vision in *A. dorsata*

*Apis dorsata* workers were trained to a blue target and tested on a stimulus target array comprising the blue and three grey targets. Most bees made the first visit to blue, although a few bees landed first on the grey targets (figure 2*c*; electronic supplementary material, table S1; $\chi_{3}^{2}=194$, *p* < 0.001, *n*_bees_ = 20); 78.9 ± 4.8% of total visits were to the blue stimulus (figure 2*d*; Friedman test $\chi_{3}^{2}=42.4$, *p* < 0.001, *n*_bees_ = 20, *n*_landings_ = 117), and the choices made to the grey stimuli were negligible (electronic supplementary material, table S2). We saw a similar performance by bees on the yellow-trained colour detection assay (electronic supplementary material, figure S2*c,d*).

*A. dorsata* discriminated the learned yellow colour during diurnal conditions from an array of four chromatic stimuli: blue, green, the learned yellow and red. All bees landed first on the yellow stimulus (figure 2*g*; $\chi_{3}^{2}=300$, *p* < 0.001, *n*_bees_ = 12), and the total proportion of choices differed significantly between colours (figure 2*h*; Friedman test $\chi_{3}^{2}=30.7$, *p* < 0.001, *n*_bees_ = 12, *n*_landings_ = 143); 88.3 ± 4.6% of all visits were to yellow, and this was significantly higher than visits made to any of the other stimuli (electronic supplementary material, table S2).

### (c) *Apis dorsata* can detect colour at night

*Apis dorsata* workers showed a strong preference to land on the blue stimulus in all light conditions and a weak preference to land first on G5.5 and G3.5 under HME and brighter conditions (greater than 0.01 cd m^−2^) (figure 3*d,g,j*; electronic supplementary material, table S1; 0.1–0.2 cd m^−2^: $\chi_{3}^{2}=128.1$, *p* < 0.001, *n*_bees_ = 11; 0.01–0.1 cd m^−2^: $\chi_{3}^{2}=174.3$, *p* < 0.001, *n*_bees_ = 11; 0.001–0.01 cd m^−2^: $\chi_{3}^{2}=28.7$, *p* < 0.001, *n*_bees_ = 17). On average, bees made five visits per trial under light levels above HME and six visits at HME, which dropped to about one visit per trial at light levels below HME. The proportion of total choices varied significantly with the colour of the stimulus and the interaction term between colour and light levels (figure 3*e,h,k*; electronic supplementary material, table S5; ANOVA: *F*_11, 140_ = 13.72, R^2^ = 0.48, *p* < 0.001). Tukey's HSD test was conducted on the best model (model 1) to estimate the difference in preference across the stimuli, light levels and the interaction term (electronic supplementary material, table S6). The performance of bees was similar when ambient light was equivalent to HME and brighter; out of the total choices made by bees, 87.5 ± 3.9% of choices were for the blue stimulus at the brightest light level (0.1–0.2 cd m^−2^, *n*_all choices_ = 56, *n*_bees_ = 11) and 76.6 ± 5.9% at HME (0.01–0.1 cd m^−2^, *n*_all choices_ = 64, *n*_bees_ = 11). This preference showed a drastic decrease below HME (0.001–0.01 cd m^−2^), with only 44.1 ± 11.2% of the choices being towards blue (*n*_all choices_ = 24, *n*_bees_ = 17), and significantly lower than their performance at the brightest light level (electronic supplementary material, table S6). The proportion of visits to the grey stimuli remained significantly lower compared with the blue stimuli across all light levels, and there was no difference in the visits made between the grey stimuli across light levels (figure 3*e,h,k*; electronic supplementary material, table S6).

## 4. Discussion

**Figure 4.**
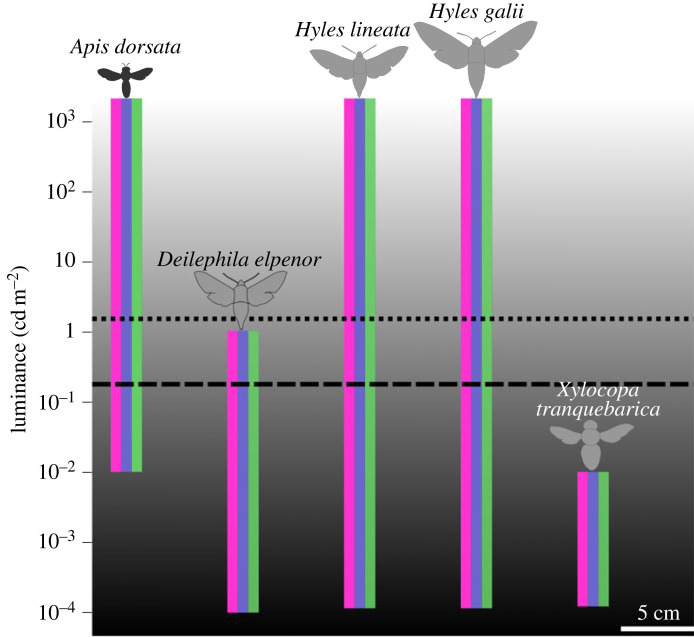
Colour vision thresholds of nocturnally active insects. The violet, blue and green bands represent trichromacy in these species. The dotted line represents the threshold at which humans switch to mesopic vision (which depends on both cones and rods), and the dashed line corresponds to the colour vision threshold in the European honeybee *Apis mellifera*. *Apis dorsata* is the first facultatively nocturnal insect with apposition eyes shown to possess nocturnal colour vision. The three hawkmoths have superposition eyes and the carpenter bee *X. tranquebarica*, with apposition eyes, is an obligately nocturnal bee. Data for this figure were sourced from references [4,7,8,10,14]. The scale bar refers to the schematic images of the five different species.

Honeybees are famously diurnal and use the sun's position for communication and navigation [59]. Most honeybee species cease foraging activity around sunset [18,35,60]. This is probably driven by the fact that their apposition eyes cannot function efficiently in dim-light. The European honeybee *Apis mellifera* cannot use colour cues at light levels comparable to half-moon intensities [32,61], and cease their activity around sunset. However, the Asian giant honeybee *Apis dorsata* [34,38] and the African honeybee *Apis mellifera adansonii* [33,62] facultatively extend foraging activity into the night [33–35,38]. While *A. dorsata* has larger eyes and ocelli compared to sympatric honeybees (*A. cerana, A. florea*) and *A. mellifera* [35], their physiological and neural adaptations for nocturnal vision, and the role of these adaptations in processing colour information in dim light, remain unknown. Although the obligately nocturnal carpenter bee, *Xylocopa tranquebarica,* can discriminate colour at starlight levels [14], we expected colour vision in *A. dorsata* to worsen in dim light given that the species is typically diurnal with nocturnal foraging restricted only to bright moonlit nights [34,35,38], and has much smaller eyes than the nocturnal *X. tranquebarica*. Here, we first show through modelling of the stimuli in bee-subjective colour spaces that the achromatic information (relative brightness) of a stimulus is an unreliable cue for Asian giant honeybees, especially under the rapidly fluctuating dim ambient light conditions when *A. dorsata* are known to forage. Secondly, we show behaviourally that bees trained to a chromatic stimulus in dim light can detect the stimulus when presented alongside grey distractor stimuli. Thus, our study establishes colour vision and robust colour learning in *A. dorsata* during the day, and remarkably, we found that this ability is maintained at half-moon light levels, making it a first in honeybees. *Apis dorsata* is thus the second insect with apposition compound eyes that possesses dim-light colour vision [1,14], the first being *X. tranquebarica* (figure 4). We report that the threshold of dim light colour vision in *A. dorsata* is surprisingly similar to humans.

The spectral classes of photoreceptors and their peak sensitivities are highly conserved in honeybees, and in most hymenopterans tested so far [27–29], possibly endowing them with comparable colour vision capabilities. It must be noted that the colour vision models we have reported consider the visual system of *Apis mellifera.* The differences in eye morphology, and the physiological adaptations shaped by ecology, might determine the limits of visual processing in *Apis dorsata* [1,35]. The models we used for twilight and moonlight, which accounted for photon shot noise, reported very low distances between all stimuli in the colour space. Photoreceptor noise and Weber fractions for *A. mellifera* used in these models could be different in the *A. dorsata* visual system. Hence, the model output cannot be used reliably to explain colour discrimination by bees under dim-light conditions. Moreover, the illuminant spectra for twilight and moonlight used in the models were not measured from the study site while the bees were being trained and tested. Although these curves are representative examples of the spectral composition, the absolute number of photons may differ considerably between locations, with weather conditions, microenvironments, exact time during twilight and with moon elevation. Additionally, quantifying the light conditions during experiments is only feasible with sensitive instruments under laboratory condition [10,63], which are quite different to the open field-like conditions we had to use in this study. Finally, colour vision models assume bright-light conditions and do not incorporate the possibility of spatial and temporal summation in dim light (see below), a mechanism that can reduce noise and thereby elevate the visual signal-to-noise ratio, considerably improving colour discrimination beyond that predicted by models [1,64]. Therefore, the outputs of the colour models, while illuminating, must be interpreted with caution.

Interestingly, nocturnal foraging in *A. dorsata* has been shown to be tightly correlated to the lunar phase, and maximum nocturnal activity has been observed during the third quarter (waning phase from full-moon to half-moon), while there was almost no activity in the first, second and fourth quarters [38]. We found similar patterns, with maximum activity occurring during the waning phase. Our results show that most *A. dorsata* workers landed first on the trained colour in both bright daylight and in dim-light conditions down to HME light intensities. Furthermore, they showed a strong preference to repeatedly land on the trained colour compared with the grey stimuli in subsequent choices as well. However, this trend did not extend to the dimmest light levels at which they were tested (between 10^−2^ and 10^−3^ cd m^−2^, corresponding to levels lower than HME), suggesting that their ability to detect chromatic cues falters at light levels below the dimmest under which they are known to forage [34,35,38].

Despite the benefits of nocturnal colour vision [1], perceiving colour at night requires adaptations to overcome noise and amplify the signal, and the superposition compound eyes which are typical of nocturnal insects (such as moths) provide a 100–1000-fold increase in sensitivity when compared with apposition eyes [65]. Not surprisingly, three out of the five insects shown to possess nocturnal colour vision are hawkmoths with superposition eyes (figure 4). Interestingly, the nocturnal carpenter bee *X. tranquebarica*—with apposition compound eyes—also uses colour cues for homing in starlight (10^−4^ cd m^−2^). All nocturnal bees studied so far show common visual morphological adaptations [8,35,66–68]. Compared to sympatric diurnal and facultatively crepuscular carpenter bees, the nocturnal carpenter bee *X. tranquebarica* has larger eyes, larger ommatidial facets and wider rhabdoms (along with larger ocelli), and this leads to increased sensitivity [9]. Similar adaptations were found in *A. dorsata* when compared to two sympatric diurnal honeybee species *Apis cerana* and *Apis florea* [35]. Although *A. dorsata* has the largest eyes (2.8 ± 0.1 mm length and 6394 facets) and ocelli (0.38 ± 0.02 mm diameter) among honeybees, they are smaller (intertegular width 3.9 ± 0.2 mm) than the nocturnal carpenter bee *X. tranquebarica* (intertegular width 8.8 ± 0.4 mm) which has larger eyes (6.7 ± 0.3 mm length and 18 804 facets) and wider ocelli (0.95 ± 0.07 mm diameter) [35,69]. While larger facets and wider rhabdoms can increase visual sensitivity in dim-light, neural adaptations like temporal and spatial summation, longer integration times and high contrast gains, are proposed to support visual behaviour in the nocturnal Panamanian sweat bee *Megalopta genalis* [68]. The same adaptations potentially permit motion vision, visual flight control and colour vision in other nocturnal bees as well [15,70–72].

There is some evidence for neural summation in dim light in other bees. For instance, the spatial resolution of *A. mellifera* in behavioural experiments at varying light intensities could not be explained by the optics and physiology of their compound eyes, and theoretical estimations that considered spatio-temporal summation at higher visual processing centres best explained the behaviour [73]. This suggests that honeybee visual systems are capable of summing visual information at the neural level, thereby enhancing visual reliability at dimmer light levels. Bumblebees (*Bombus terrestris*) maintain flight performance at lower light levels by flying more slowly and having a reduced photoreceptor response speed, both of which are suggestive of temporal summation [74]. It is interesting to note that, at least in the context of homing, earlier experiments suggested that the neotropical *M. genalis* is unable to learn colour at night, although recent preliminary experiments are beginning to suggest otherwise (EJ Warrant 2023, personal communication). Nonetheless, we can now with certainty establish nocturnal colour vision in two palaeotropical Asian bees: *A. dorsata* and *X. tranquebarica* [14]. It is still too early to speculate on any species-specific and environment-specific differences, and further studies are required from other bee species and habitats. We also noticed that any changes to the set-up at night immediately demotivated the bees, so we trained and tested the bees to larger stimuli resembling the training feeder instead of the flat paper discs that we used in the diurnal experiments. This also suggests that the salience of a stimulus at night could depend on other visual factors such as the shape and size of the floral display, in addition to colour.

Since the ambient illumination in the two experimental locations where we did the nocturnal experiments on *A. dorsata* was never less than 10^−2^ cd m^−2^ (which corresponds to half-moon light levels), we used an experimental arena made of a cardboard box lined with black chart paper to reduce the light levels to approximately 10^−3^ cd m^−2^ inside the box. The apparent reluctance of bees to make choices in this arena, as well as their reduced ability to discriminate colour at the dimmest light levels at which we tested bees (10^−3^–10^−2^ cd m^−2^), may be due to the sudden drop in light intensities experienced by the bees when they enter the darker arena from a brighter exterior (where the illumination was greater than 10^−2^ cd m^−2^). This sudden change in light levels, not immediately matched to the adaptation state of their photoreceptors, nor to their present extent of neural summation (if present), could reduce the performance of bees in tests. Indeed, our observations suggest that decision speed was considerably slower and that bees usually made only a single (and often incorrect) choice when landing on a stimulus target. At higher light levels, the bees predominantly visited the blue stimulus over the course of the trial. Even on those rare occasions when they landed on one of the greys, they subsequently visited blue. However, bees rarely made multiple choices at the dimmest light condition (unlike under brighter conditions), indicating a significant reduction in their ability to discriminate colour. Even though their performance suffered at light levels below HME, 45% of first choices were still towards the trained blue stimulus (figure 3*j*) suggesting that colour vision might be robust in some bees even at such low light levels (10^−3^ cd m^−2^, a light level lower than HME), although they are not reported to naturally forage on flowers below half-moon light levels.

*A. dorsata* increasingly nests in urban areas, as high-rise buildings provide stable nesting substrates and the potential availability of diverse resources from urban green spaces (such as gardens) year-round [38,39,41,75]. In addition to this, the introduction and increased use of white-LED lights is causing wider shifts in the illumination spectrum at night [1]. It is hence important to consider the nocturnal habits of *A. dorsata* in the context of foraging in an increasingly light-polluted habitat [44]. In our tests, the bees continued to forage and make accurate choices even with considerable skyglow. However, skyglow was not sufficient to elicit foraging activity in *A. dorsata* and it depended on the moon phase (figure 1*c*). Whether light pollution disrupts the foraging ecology of these bees, and impacts their nocturnal pollination, remains unexplored. Studies comparing the nocturnal activity of these bees in light-polluted urban areas with bees and in light-pollution-free areas (such as forests) can shed light on their nocturnal foraging ecology and potential role in pollination. In urban areas, even though their activity is not initiated (or maintained beyond sunset) by artificial lights, foraging might be aided by lights in gardens and green spaces and can potentially impact the onset, termination, range and distance of foraging trips in different habitats [76].

Interestingly, the natural colonies from IISER-TVM which were used for the diurnal experiments did not exhibit any nocturnal foraging activity across 2 years. Since this site is located in the foothills of the southern Western Ghats and shares borders with tropical moist deciduous and semi-evergreen forests, as well as home gardens and plantations, it is reasonable to expect that the colonies are not limited by floral resources. This is indicated by foragers returning with pollen year-round during daytime, even during the rainy season (authors' personal observations, 2019–2021). As the resources are abundant and diurnal foraging is enough to accrue sufficient resources, *A. dorsata* might not extend foraging into the night, even if light levels are sufficiently bright to initiate flight activity. Another factor that might prevent nocturnal activity could be the dimmer conditions under the forest canopy. The light levels deteriorate quickly under canopy cover and might make navigation difficult [68,77,78]. In Bhimashankar wildlife sanctuary, in the northern Western Ghats, *A. dorsata* was observed to limit their nocturnal foraging activity to open areas and to the edges of forests [35]. Nocturnal foraging in *A. dorsata* was also reported from dry deciduous forest habitats in Koundinya wildlife sanctuary in the Eastern Ghats (K Mohan 2018, personal communication), which are mainly thorn forests and shrub thickets with a sparse canopy cover. While the IISER-TVM campus had both artificial light sources as well as green spaces, neither factor induced nocturnal foraging in *A. dorsata* colonies. Hence, we assume that site-specific differences in temporal foraging dynamics, as discussed here, are mostly driven by resource abundance and availability in this facultatively nocturnal bee.

Nocturnality in bees is atypical and has evolved independently in several families [79]. The evolution of dim-light vision in bees is proposed to be driven by the advantages in shifting the temporal niche to exploit night-blooming flowers [79] while avoiding competition, predation [80–82] and high temperatures [5,60]. However, in *A. dorsata*, nocturnality is facultative and limited by ambient illumination. While resource-dependence of nocturnal foraging in *A. dorsata* has been reported previously [38], the role of other factors is not known. Although the nocturnal pollination services of honeybees are yet to be well-characterized, there is considerable evidence for nocturnal crop pollination by bees [83], in addition to moths (Sphingidae and Noctuidae) and bats (Phyllostomidae) [84]. Furthermore, the truly nocturnal carpenter bee *X. tranquebarica* forages mostly on day-blooming flowers that remain open at night and is reported to be less flower-constant at lower light levels. This is not ideal from the plants’ perspective as it limits conspecific pollen transfer and may cause stigma clogging [85]. *Apis dorsata* flies at brighter light levels than *X. tranquebarica*, and whether this affects floral constancy, and which role (if any) *A. dorsata* plays in nocturnal pollination, remain to be explored. Furthermore, it is not known whether artificial light and skyglow affects their foraging range and ecology. It would be interesting to address these in urban and forest-dwelling colonies and to compare their pollination efficiencies at night. Such studies will help us better understand the effect of anthropogenic stressors on ecosystem services.

Finally, but just as importantly, the physiological and neural adaptations that enable dim-light colour vision remain to be explored in *A. dorsata*, as do the roles of multi-sensory integration in finding flowers and whether they actually improve floral colour discrimination at night. With robust colour vision at light levels at which the European honeybee cannot see colour, *A. dorsata* is an ideal system to address the evolution of nocturnality and the limits of visual performance and processing in insects.

## Data Availability

The data and code used in this study are given in the electronic supplementary material [86].
